# A nationwide comparison of epidemiologic characteristics and outcomes between intentional and unintentional severe external-cause injuries in Korea, 2016–2020

**DOI:** 10.1186/s40621-026-00701-5

**Published:** 2026-07-16

**Authors:** Jeongho Oh, Han Zo Choi, Jongkyeong Kang, Sook-Young Woo, Seung Jin Maeng

**Affiliations:** 1 Department of Emergency Medicine, Kyung Hee University Hospital at Gangdong, Seoul, Republic of Korea; 2 Department of Statistics, Kangwon National University, Chuncheon, Republic of Korea; 3 Department of Big Data and AI, Daejeon University, Daejeon, Republic of Korea; 4 Department of Emergency Medicine, Samsung Medical Center, Seoul, Republic of Korea

**Keywords:** Intentional injury, Unintentional injury, Severe injury, External-cause injury, Emergency medical services, Injury surveillance, Korea

## Abstract

**Background:**

Intent-based injury surveillance requires distinction between injury intent and mechanism, particularly when datasets include anatomical trauma and non-traumatic external-cause injuries such as drug intoxication. This study compared mechanisms, incidence, regional variation, transfer pathways, and short-term outcomes between intentional and unintentional severe external-cause injuries in South Korea.

**Methods:**

This retrospective nationwide study used the Korea Disease Control and Prevention Agency Community-Based Severe Trauma Survey (2016–2020). The source population comprised patients with severe trauma, non-traumatic severe injury, or mass casualty incidents transported to emergency departments by 119 Emergency Medical Services and confirmed through hospital record review. Intentional injury was defined as self-harm/suicide or violence/homicide, and unintentional injury as recorded unintentional injury. Drug intoxication was treated as a mechanism, not as intent. Descriptive analyses used the full cohort, whereas primary multivariable models used the prespecified non-intoxication cohort. Age-specific incidence and mortality were calculated as pooled 2016–2020 rates using summed annual population denominators. Logistic regression estimated adjusted odds ratios (aORs) for emergency department (ED) and post-admission survival.

**Results:**

Among 230,302 transported cases, 55,513 were intentional and 174,789 were unintentional; 45,093 involved drug intoxication. Intentional injuries were more commonly associated with drug intoxication and hanging or strangulation, whereas unintentional injuries were mainly transportation- or fall-related. Out-of-hospital cardiac arrest was more frequent after intentional injuries (21.8% vs. 9.8%), as were ED death (19.6% vs. 9.8%) and documented ED or in-hospital mortality after ED presentation (23.3% vs. 13.7%). In the non-intoxication cohort, intentional injury was associated with lower ED survival (aOR, 0.215; 95% CI, 0.207–0.222) and lower post-admission survival (aOR, 0.561; 95% CI, 0.521–0.604). Age-specific incidence and mortality were generally higher for unintentional injuries, whereas transported-case fatality was higher for intentional injuries.

**Conclusions:**

Intentional and unintentional external-cause injuries differed in mechanism distribution, prehospital deterioration, age-specific burden, transfer pathways, and outcomes. Drug intoxication should be distinguished from injury intent and anatomical trauma. These findings support clear intent-mechanism classification and targeted prevention and emergency care strategies.

**Supplementary Information:**

The online version contains supplementary material available at https://doi.org/10.1186/s40621-026-00701-5.

## Introduction

According to the World Health Organization (WHO), injury encompasses physical damage resulting from acute exposure to mechanical, thermal, electrical, or chemical energy (e.g., poisoning), or from the absence of essentials such as oxygen (e.g., asphyxiation) [1]. Injury is commonly classified according to the underlying intent of the event, most broadly as unintentional or intentional [2]. This distinction is not merely descriptive; it reflects differences in mechanisms of occurrence, risk profiles, prevention targets, healthcare needs, and clinical trajectories after emergency medical services (EMS) activation [2, 3]. Unintentional injuries are commonly related to transportation incidents, falls, occupational injuries, drowning, burns, and accidental poisoning, whereas intentional injuries include self-directed harm, self-poisoning, interpersonal violence, and other deliberately inflicted injuries [3]. Accordingly, intent-based classification provides an important framework for injury surveillance, prevention policy, EMS triage, and emergency care planning [1, 4]. 

Injuries remain a major cause of death and disability worldwide and impose a substantial burden on emergency medical systems [5]. Because the mechanisms and clinical courses of injuries differ according to intent, understanding the epidemiologic and outcome differences between intentional and unintentional injuries is essential for developing targeted prevention strategies and improving acute care systems [2, 4]. This issue is particularly relevant in South Korea, which consistently reports the highest suicide rate among Organization for Economic Co-operation and Development (OECD) member countries, magnifying the systemic burden of intentional injuries on acute care systems [6]. Consequently, injury-related mortality and healthcare utilization remain critical public health concerns across the life course, including adolescents, young adults, and older adults [7]. In addition, intentional injuries may present with distinct prehospital and emergency department (ED) characteristics, including delayed discovery, physiological deterioration before medical contact, different lethal mechanisms such as self-poisoning or asphyxiation, and greater need for critical care or psychiatric intervention [2, 3]. 

Previous studies in South Korea have described the epidemiology of traumatic injuries, the predominance of transportation-related injuries and falls, and the survival benefit associated with treatment at regional trauma centers [8–10]. Other studies comparing intentional and unintentional injuries have focused mainly on children, adolescents, psychiatric risk factors, or selected communities, emphasizing socioeconomic, environmental, and behavioral determinants [11–13]. Although nationwide community-based reports have characterized the overall incidence and outcomes of severe injury in South Korea [14, 15], evidence directly comparing intentional and unintentional severe external-cause injuries across all age groups remains limited. In particular, few studies have comprehensively evaluated early ED outcomes, post-admission outcomes, inter-hospital transfer patterns, and regional variation according to injury intent using a nationwide EMS-based dataset.

An important methodological consideration is that the Community-Based Severe Trauma Survey is not limited to anatomically defined trauma. The survey includes KDCA-defined EMS-transported severe trauma, non-traumatic severe injury, and mass casualty cases confirmed through hospital medical record review [16, 17]. Therefore, selected non-traumatic external-cause mechanisms, including drug intoxication, are captured within the survey framework. Although poisoning and intoxication are highly relevant external-cause events for injury surveillance and prevention, they differ fundamentally from anatomical trauma in pathophysiology, clinical management, and the interpretation of anatomical severity scores such as the Injury Severity Score [18]. Accordingly, analyses that include intoxication can provide a broad public health perspective on EMS-transported severe external-cause injuries, whereas analyses excluding intoxication are necessary to determine whether the observed associations are consistent among patients with non-intoxication injuries.

Comparing intentional and unintentional severe external-cause injuries is also relevant beyond the Korean setting. Many healthcare systems face similar challenges related to aging populations, self-harm and suicide prevention, regional disparities in emergency care access, and the integration of prehospital triage with trauma and emergency medical systems [4–6]. A nationwide EMS-based analysis from South Korea may therefore provide useful evidence for other systems seeking to strengthen injury surveillance, optimize acute care pathways, and develop intent-specific prevention and intervention strategies.

Accordingly, this study aimed to compare the epidemiologic characteristics, injury mechanisms, incidence patterns, regional variation, inter-hospital transfer patterns, and short-term clinical outcomes of intentional and unintentional severe external-cause injuries using nationwide data from the Community-Based Severe Trauma Survey conducted by the Korea Disease Control and Prevention Agency between 2016 and 2020 [16, 17]. We further evaluated the association between injury intent and survival in the ED and after hospital admission. Given the distinct clinical characteristics of drug intoxication, we also performed prespecified analyses excluding intoxication cases and separately described the characteristics and outcomes of patients with intoxication.

## Methods

### Ethics statement

This study was approved by the Institutional Review Board of KyungHee University Hospital at Gangdong (IRB No. 2025-11-055), and it adhered to the ethical guidelines of the 1975 Declaration of Helsinki. Due to the retrospective nature of the study and the use of de-identified data, the requirement for informed consent was waived by the Institutional Review Board.

### Study design, data source, and setting

This retrospective nationwide study used data from the Community-Based Severe Trauma Survey conducted by the Korea Disease Control and Prevention Agency (KDCA) between 2016 and 2020 [16, 17]. According to the KDCA raw data use guide, the survey target population was operationally defined as patients with severe trauma, non-traumatic severe injury, or mass casualty incidents who were transported to emergency departments by the Korean 119 Emergency Medical Services (EMS) and confirmed through structured hospital medical record review [17]. Therefore, the survey represents a nationwide EMS-based complete survey within a defined target population, rather than a registry of all injured patients in the community. Patients with minor injuries, those transported by private vehicles or private ambulances, and those who died at the scene without EMS transport were not captured in this dataset. In this manuscript, the term “severe” refers to inclusion in the KDCA survey target population of EMS-transported cases confirmed through the survey framework, and should not be interpreted as indicating that all included patients had anatomically severe trauma defined by ISS ≥ 16.

The KDCA survey framework includes three major source categories: severe trauma, non-traumatic severe injury, and mass casualty incidents [16, 17]. This distinction is important because the source dataset is not limited to anatomically defined trauma. Non-traumatic severe injury mechanisms include external-cause mechanisms such as thermal injury, asphyxial mechanisms, drowning, and contact with chemical or other substances. In the present analytic classification, drug intoxication was treated as a non-traumatic external-cause injury mechanism rather than as a definition of intentional injury. Accordingly, the present study should be interpreted as a comparison of KDCA-defined EMS-transported severe external-cause injuries classified by recorded injury intent.

### Injury intent, mechanism, and severity

Patients were classified into intentional and unintentional injury groups according to the injury intent variable recorded in the KDCA raw data use guide. Intentional injury was defined as cases coded as self-harm/suicide or violence/homicide, whereas unintentional injury was defined as cases coded as unintentional injury. Cases coded as other or unknown intent were excluded from the intentional-versus-unintentional comparison.

Injury intent was conceptually distinguished from injury mechanism. Injury mechanism described the external cause or clinical mechanism of injury and included both traumatic and non-traumatic mechanisms, including drug intoxication. Drug intoxication was therefore treated as an injury mechanism rather than as a definition of intentional injury, because intoxication cases could be classified as either intentional or unintentional depending on the recorded circumstances of the event.

Injury severity was assessed using the Injury Severity Score (ISS), which is calculated from the Abbreviated Injury Scale [19, 20]. Valid anatomical ISS values were defined as ISS values ranging from 1 to 75. ISS codes 88 and 99, indicating OHCA or prehospital death and unknown ISS, respectively, were not treated as continuous anatomical severity scores and were excluded only from analyses that modeled ISS as a continuous covariate [17]. When ISS was presented as a continuous descriptive variable or modeled as a continuous covariate, it was restricted to valid anatomical ISS values from 1 to 75. Because drug intoxication is a toxicologic mechanism that is not adequately represented by anatomical ISS, intoxication cases were described separately and excluded from the prespecified non-intoxication multivariable survival analyses. The primary multivariable models were fitted in the non-intoxication cohort without adjustment for ISS, whereas secondary ISS-adjusted models were further restricted to patients with valid anatomical ISS values from 1 to 75.

### Analytic cohorts and outcomes

**Fig. 1:**
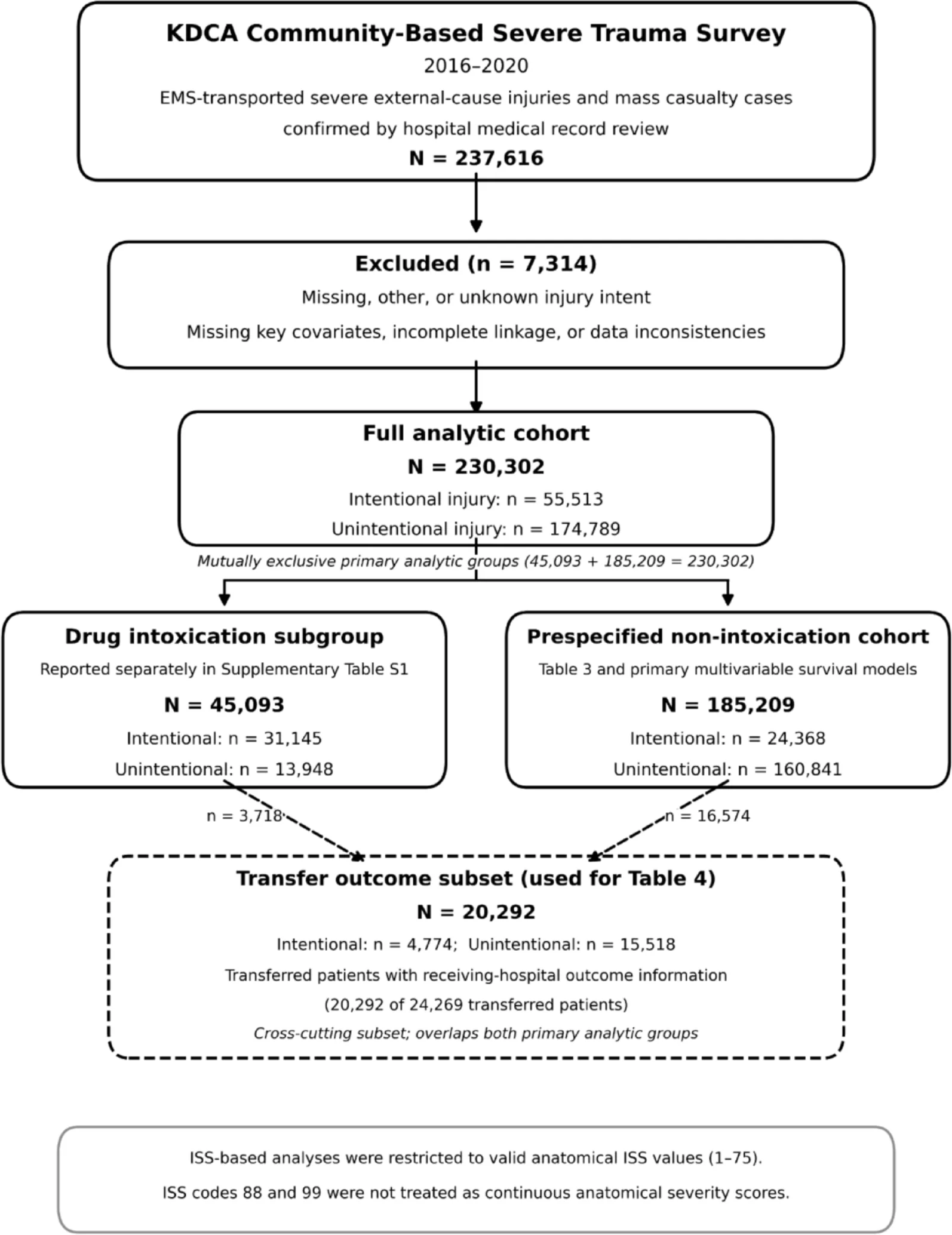
Flow diagram of study population selection and analytic cohorts

The analyses were structured according to the study objectives and the analytic cohorts shown in Fig. 1. The full external-cause injury cohort was used for descriptive comparisons of demographic characteristics, injury mechanisms, prehospital factors, hospital level, treatment interventions, and clinical outcomes according to recorded injury intent. Drug intoxication cases were summarized separately as a prespecified subgroup in Supplementary Table S1. A prespecified non-intoxication cohort was used for Table 3 and for the main multivariable survival models. Table 3 was used to describe the non-intoxication cohort after excluding drug intoxication cases, whereas the regression models used model-specific complete-case samples according to the availability of the outcome and covariates included in each model. For the inter-hospital transfer analysis, Table 4 was restricted to patients transferred from the initial ED to another institution who had available receiving-hospital outcome information. This transfer analysis was intended to describe transfer pathways and receiving-hospital outcomes among patients with ascertainable receiving-hospital outcome data, rather than to estimate causal effects of transfer. In this transfer subset, we summarized injury mechanism distribution, reasons for transfer, ED disposition at the receiving hospital, ICU admission among admitted patients, post-admission outcomes, and overall outcomes after presentation to the receiving hospital according to recorded injury intent.

ED survival was defined as discharge from the ED, transfer to another hospital, or hospital admission, whereas death in the ED was defined as non-survival at the ED stage. Post-admission survival was defined as survival to discharge among patients admitted through the ED, whereas in-hospital death after admission was defined as non-survival after admission. Overall mortality after ED presentation was defined using documented ED death or in-hospital death after admission in the survey record; deaths occurring after transfer to another institution were not captured in this index-hospital measure. For transferred patients in Table 4, ED disposition, post-admission outcomes, and overall outcomes were defined after presentation to the receiving hospital among patients with available receiving-hospital outcome information.

The source survey population included KDCA-defined EMS-transported severe external-cause injury and mass casualty cases confirmed through hospital medical record review. Patients with missing, other, or unknown injury intent, missing key covariates, incomplete hospital linkage, or data inconsistencies were excluded from the full analytic cohort. Drug intoxication cases and non-intoxication cases formed mutually exclusive primary analytic groups. Drug intoxication cases were summarized separately in Supplementary Table S1, whereas the prespecified non-intoxication cohort was used for Table 3 and the primary multivariable survival models. The transfer outcome subset was a cross-cutting subset of transferred patients with available receiving-hospital outcome information and overlapped both primary analytic groups. ISS-based analyses were restricted to valid anatomical ISS values ranging from 1 to 75; ISS codes 88 and 99 were not treated as continuous anatomical severity scores. Patients who died at the scene without EMS transport were outside the survey population.

### Statistical analysis

Continuous variables were presented as means with standard deviations or medians with interquartile ranges, as appropriate. Categorical variables were presented as numbers and percentages. Student’s t-test or the Mann–Whitney U test was used for continuous variables, and the chi-square test was used for categorical variables.

Age-specific incidence rates were calculated as pooled 2016–2020 rates per 100,000 person-years. For each age group and injury-intent category, the numerator was the total number of EMS-transported severe external-cause injury cases during 2016–2020, and the denominator was the sum of the corresponding annual resident population counts for that age group during the same 5-year period. The summed annual resident population counts were treated as an approximation of total person-years at risk. Thus, these estimates were calculated using 5-year summed population denominators, not 5-year average population denominators. Age-specific case counts were presented as absolute numbers of transported cases during 2016–2020. Age-specific case fatality was calculated as the number of deaths divided by the number of transported cases within each age group and injury-intent category. Because case-fatality estimates may be unstable in subgroups with small numbers of transported cases, especially in younger age strata with few intentional injury cases, these subgroup-specific fatality estimates were interpreted descriptively. Age-specific mortality rates were calculated as pooled 2016–2020 mortality rates per 100,000 person-years, using the total number of deaths during 2016–2020 divided by the same summed annual age-specific population denominator and multiplied by 100,000. Population denominators were obtained from Statistics Korea [21]. These age-specific incidence and mortality rates were not age-standardized and should be interpreted as the burden among KDCA-defined EMS-transported severe external-cause injuries, rather than as the incidence or mortality of all injuries in the community.

For regional analyses, crude regional rate ratios were calculated by dividing each region’s crude incidence or mortality rate by the national pooled crude rate during 2016–2020. The national pooled rate was used as the reference value of 1.0, and regional estimates were not age- or sex-standardized. Regional rate ratios were therefore interpreted as descriptive crude regional comparisons rather than adjusted regional effects.

Multivariable logistic regression models were constructed to estimate adjusted odds ratios for ED survival and post-admission survival. The primary exposure variable was recorded injury intent, with unintentional injury used as the reference group. The primary models excluded drug intoxication cases and were adjusted for age group, sex, insurance status, broad injury mechanism category, and hospital level. Insurance status was categorized as medical insurance, Medicaid, or other insurance, with medical insurance used as the reference category. Broad injury mechanism category was classified as traumatic versus non-traumatic injury, with traumatic injury used as the reference category. Hospital level was categorized as trauma center, regional emergency medical center, local emergency medical center, or emergency medical center, with trauma center used as the reference category. Because recorded injury intent and broad mechanism category were structurally related, mechanism-specific coefficients were treated as adjustment terms and were not interpreted as independent causal effects.

The primary models did not include ISS or prehospital physiological variables. Secondary ISS-adjusted models were restricted to patients with valid anatomical ISS values from 1 to 75 and additionally included ISS as a continuous covariate. Prehospital physiological variables, including vital signs, mental status, and OHCA, were not included in the primary or ISS-adjusted secondary models because they may represent downstream physiological deterioration after the injury event rather than baseline confounding factors [22]. Sensitivity models additionally adjusting for OHCA were performed to evaluate the robustness of the association between recorded injury intent and survival. The OHCA-adjusted sensitivity models used the same modeling strategy as the corresponding primary models, with prehospital OHCA added as an additional covariate. Because prehospital OHCA is closely linked to ED death and may induce sparse-data or separation-related instability, OHCA-adjusted models were interpreted only as sensitivity analyses rather than as primary explanatory models.

Baseline and descriptive comparisons were intended to describe distributional differences rather than to test a single confirmatory hypothesis. Therefore, formal adjustment for multiple comparisons was not applied. Standardized mean differences were calculated to aid interpretation of between-group differences, and an SMD greater than 0.1 was considered to indicate a potentially meaningful imbalance [23]. 

Because the proportion of excluded cases due to missing key covariates, incomplete linkage, data inconsistencies, or missing/other/unknown injury intent was small (7,314 of 237,616 patients, 3.1%), complete-case analyses were performed without imputation. For multivariable analyses, complete-case samples were defined separately for each model according to the availability of the outcome and covariates included in that model; therefore, model-specific sample sizes were reported in each regression table. All statistical analyses were performed using R software, version 4.3.1 (R Foundation for Statistical Computing, Vienna, Austria). P-values were based on a two-sided significance level of 0.05, and multicollinearity was assessed using variance inflation factors.

## Results

### Study population and analytic cohorts

Between 2016 and 2020, a total of 237,616 KDCA-defined EMS-transported severe external-cause injury and mass casualty cases were confirmed through structured hospital medical record review. Among these, 7,314 patients were excluded because of missing, other, or unknown injury intent, missing key covariates, incomplete linkage, or data inconsistencies. The final full analytic cohort consisted of 230,302 patients, including 55,513 patients classified as having intentional injuries and 174,789 patients classified as having unintentional injuries.

Drug intoxication accounted for 45,093 cases in the full cohort, including 31,145 intentional and 13,948 unintentional cases. Because drug intoxication differs from other injury mechanisms in pathophysiology and in the interpretation of anatomical severity scores, these patients were summarized separately in Supplementary Table S1. After excluding drug intoxication cases, the non-intoxication cohort consisted of 185,209 patients, including 24,368 intentional and 160,841 unintentional injury cases. This cohort was used for Table 3 and for the multivariable logistic regression analyses of ED survival and post-admission survival.

### Full-cohort patient and prehospital characteristics

Table 1 summarizes the demographic and prehospital characteristics of the full analytic cohort. Mean age was similar between groups, and intentional injuries were less often male than unintentional injuries. Medical insurance was less common in the intentional injury group, whereas Medicaid and other insurance types (including self-pay and automobile insurance) were more common (Table 1).

The mechanism distribution showed the largest between-group difference in the full cohort (SMD = 2.292). Intentional injuries were more frequently associated with non-traumatic mechanisms, particularly drug intoxication (56.1%) and hanging or strangulation (13.1%), whereas unintentional injuries were predominantly traumatic, with transportation incidents (49.6%) and falls or slip-down injuries (29.3%) accounting for most cases.

Prehospital transport time and hospital level showed only small between-group differences (Table 1). OHCA was more frequent among patients with intentional injuries (21.8% vs. 9.8%).

**Table 1:** Patient and prehospital characteristics of EMS-transported severe external-cause injuries according to recorded injury intent (*N* = 230,302)

	Intentional (*N* = 55,513)	Unintentional (*N* = 174,789)	*P*-value	SMD
**Age, years (Mean ± SD)**	48.4 ± 18.7	49.8 ± 22.8	< 0.001	0.067
**Sex, N (%)**			< 0.001	0.298
Male	28,000 (50.4)	113,619 (65.0)		
Female	27,513 (49.6)	61,170 (35.0)		
Insurance, N (%)			< 0.001	0.339
Medical insurance	45,221 (81.5)	161,468 (92.4)		
Medicaid	5,231 (9.4)	8,605 (4.9)		
Others (e.g., self-pay, automobile insurance)	5,061 (9.1)	4,716 (2.7)		
**Mechanism of injury, N (%)**			< 0.001	2.292
**Traumatic injury**				
Transportation incidents	93 (0.2)	86,779 (49.6)		
Falls / Slip down	8,201 (14.8)	51,142 (29.3)		
Blunt trauma	3,847 (6.9)	6,222 (3.6)		
Stab / Penetrating trauma	4,133 (7.4)	2,263 (1.3)		
Machine-related injury	7 (0.0)	2,516 (1.4)		
**Non-traumatic injury**				
Thermal injury	83 (0.1)	2,953(1.7)		
Hanging / Strangling	7,285 (13.1)	145 (0.1)		
Drowning / Suffocation	634 (1.1)	4,924 (2.8)		
Drug intoxication	31,145 (56.1)	13,948 (8.0)		
Others	18 (0.0)	1,800 (1.0)		
Missing	67 (0.1)	2,097 (1.2)		
**Time taken from 119 call to the ED arrival, minutes (Mean ± SD)**	37.6 ± 29.5	41.9 ± 32.6	< 0.001	0.138
**Level of hospital, N (%)**			< 0.001	0.185
Trauma center	9,050 (16.3)	30,179 (17.3)		
Regional EMC	11,809 (21.3)	31,496 (18.0)		
Local EMC	25,846 (46.6)	73,617 (42.1)		
EMC	8,808 (15.9)	39,497 (22.6)		
**Pre-hospital OHCA, N (%)**			< 0.001	0.332
OHCA	12,122 (21.8)	17,209 (9.8)		
Non OHCA	43,391 (78.2)	157,580 (90.2)		

ED = Emergency department; SD = Standard deviation;

OHCA = Out of hospital Cardiac Arrest; EMC = Emergency medical center

SMD = Standardized mean difference

### Full-cohort ED and post-admission outcomes

Table 2 presents ED and post-admission outcomes in the full analytic cohort. Death in the ED was more frequent among patients with intentional injuries (19.6% vs. 9.8%), and survival to discharge directly from the ED was less common (32.6% vs. 44.1%); survival to transfer and to admission were similar between groups. Among admitted patients, ICU admission was more frequent in the intentional injury group, whereas surgery or transcatheter arterial embolization was less frequently performed (Table 2). Survival to discharge after admission was similar between groups. However, when ED deaths were combined with post-admission outcomes, documented overall mortality after ED presentation was higher in the intentional injury group (23.3% vs. 13.7%).

**Table 2:** Emergency department and post-admission outcomes of EMS-transported severe external-cause injuries according to recorded injury intent (*N* = 230,302)

	Intentional (*N* = 55,513)	Unintentional (*N* = 174,789)	*P*-value	SMD
**Outcome in the ED, N (%)**			< 0.001	0.323
Survival-to-discharge	18,110 (32.6)	77,015 (44.1)		
Survival-to-transfer	5,633 (10.1)	18,636 (10.7)		
Survival-to-admission	20,867 (37.6)	62,028 (35.5)		
Death in the ED	10,903 (19.6)	17,110 (9.8)		
**ICU admission among** **admitted patients, N (%)**	14,940 (71.6)	31,159 (50.2)	< 0.001	0.449
**Surgery or TAE performed** **among admitted patients, N (%)**	3,304 (15.8)	27,775 (44.8)	< 0.001	0.664
**Time taken from the ED to surgery, hours (Median (IQR))**	7.6 (2.3–43.9)	18.4 (4.1–95)	< 0.001	0.146
**Time taken from the ED to TAE, hours (Median (IQR))**	2.8 (1.9–4.7)	2.7 (1.7–4.6)	0.224	0.106
**Result after admission, N (%)**	(*N* = 20,867)	(*N* = 62,028)	< 0.001	0.036
Survival to discharge	18,818 (90.2)	55,266 (89.1)		
Death	2,049 (9.8)	6,762 (10.9)		
**Result after ED, N (%)**			< 0.001	0.252
Survival	36,928 (66.5)	132,281 (75.7)		
Death	12,952 (23.3)	23,872 (13.7)		
Transfer to other institution	5,633 (10.1)	18,636 (10.7)		

ED = Emergency department; ICU = Intensive care unit;

TAE = Trans-catheter arterial embolization

SMD = Standardized mean difference

### Drug intoxication subgroup

The characteristics and outcomes of patients with drug intoxication are presented in Supplementary Table S1. Among patients with drug intoxication, intentional cases were more frequently female than unintentional cases and more frequently had Medicaid coverage. OHCA was uncommon in both intentional and unintentional intoxication cases. ED disposition differed between the two groups, with intentional intoxication cases showing lower survival to discharge directly from the ED and higher survival to admission. Among admitted patients, ICU admission was more frequent in the intentional intoxication group. Overall survival after ED presentation was lower in the intentional intoxication group than in the unintentional intoxication group.

### Non-intoxication cohort

Table 3 presents the characteristics and outcomes of the non-intoxication cohort. After excluding drug intoxication, the intentional group remained slightly younger, the sex distribution was similar between groups, and insurance status continued to differ, with Medicaid and other insurance types more common in the intentional group (Table 3).

The mechanism distribution remained markedly different after exclusion of drug intoxication (SMD = 2.146). In the intentional injury group, common mechanisms included falls or slip-down injuries (33.7%), hanging or strangulation (29.9%), stab or penetrating trauma (17.0%), and blunt trauma (15.8%), whereas unintentional injuries remained predominantly transportation-related (54.0%) or fall-related (31.8%).

Prehospital transport time and hospital level differed only modestly between groups (Table 3). OHCA was markedly more frequent in the intentional injury group (45.0% vs. 10.4%; SMD = 0.836), and death in the ED was also more frequent among intentional injuries (39.6% vs. 10.5%), whereas survival to ED discharge and to admission were less common.

Despite these marked differences in OHCA and ED mortality, ISS values were similar between groups (median 5 [IQR 1–10] vs. 4 [IQR 1–10]; SMD = 0.042), and this anatomical ISS summary should not be read as a measure of toxicologic or physiologic severity. Among admitted patients, ICU admission was more frequent in the intentional group, whereas surgery or transcatheter arterial embolization was performed at nearly identical frequencies (47.3% vs. 46.8%; Table 3).

Post-admission survival was lower in the intentional injury group (78.9% vs. 88.8%), and overall mortality after ED presentation was substantially higher (45.4% vs. 14.6%; Table 3).

**Table 3:** Characteristics and outcomes of the non-intoxication severe external-cause injury cohort according to recorded injury intent (*N* = 185,209)

	Intentional (*N* = 24,368)	Unintentional (*N* = 160,841)	*P*-value	SMD
**Age, years (Mean ± SD)**	46.8 ± 19.0	50.0 ± 22.9	< 0.001	0.152
**Sex, N (%)**			< 0.001	0.091
Male	15,023 (61.7)	106,221 (66.0)		
Female	9,345 (38.3)	54,620 (34.0)		
**Insurance, N (%)**			< 0.001	0.397
Medical insurance	19,528 (80.1)	148,792 (92.5)		
Medicaid	1,918 (7.9)	7,774 (4.8)		
Others	2,922 (12.0)	4,275 (2.7)		
**Mechanism of injury, N (%)**			< 0.001	2.146
**Traumatic injury**				
Transportation incidents	93 (0.4)	86,779 (54.0)		
Falls / Slip down	8,201 (33.7)	51,142 (31.8)		
Blunt trauma	3,847 (15.8)	6,222 (3.9)		
Stab/Penetrating trauma	4,133 (17.0)	2,263 (1.4)		
Machine-related injury	7 (0.0)	2,516 (1.6)		
**Non-traumatic injury**				
Thermal injury	83 (0.3)	2,953 (1.8)		
Hanging/Strangling	7,285 (29.9)	145 (0.1)		
Drowning/Suffocation	634 (2.6)	4,924 (3.1)		
Others	18 (0.1)	1,800 (1.1)		
Missing	67 (0.3)	2,097 (1.3)		
**Time taken from 119 call to the ED arrival, minutes (Mean ± SD)**	35.5 ± 30.4	40.9 ± 31.7	< 0.001	0.174
**Level of hospital, N (%)**			< 0.001	0.093
Trauma center	4,322 (17.7)	28,839 (17.9)		
Regional EMC	4,768 (19.6)	29,089 (18.1)		
Local EMC	10,786 (44.3)	67,545 (42.0)		
EMC	4,492 (18.4)	35,368 (22.0)		
**Pre-hospital OHCA, N (%)**			< 0.001	0.836
OHCA	10,958 (45.0)	16,793 (10.4)		
Non OHCA	13,410 (55.0)	144,048 (89.6)		
**Outcome in the ED, N(%)**			< 0.001	0.723
Survival-to-discharge	6,028 (24.7)	66,868 (41.6)		
Survival-to-transfer	1,989 (8.2)	17,895 (11.1)		
Survival-to-admission	6,690 (27.5)	59,249 (36.8)		
Death in the ED	9,661 (39.6)	16,829 (10.5)		
**ICU admission among** **admitted patients, N (%)**	4,953 (74.0)	30,042 (50.7)	< 0.001	0.496
**ISS (Median (IQR))**	5 (1–10)	4 (1–10)	< 0.001	0.042
**Surgery or TAE performed** **among admitted patients, N (%)**	3,164 (47.3)	27,740 (46.8)	0.460	0.010
**Time taken from the ED to surgery, hours (Median (IQR))**	6.9 (2.2–37.9)	18.4 (4.1–95.1)	< 0.001	0.220
**Time taken from the ED to TAE,** **hours (Median (IQR))**	2.8 (1.9–4.6)	2.7 (1.7–4.5)	0.289	0.126
**Result after admission, N (%)**	(*N* = 6,690)	(*N* = 59,249)	< 0.001	0.271
Survival to discharge	5,277 (78.9)	52,592 (88.8)		
Death	1,413 (21.1)	6,657 (11.2)		
**Result after ED, N (%)**			< 0.001	0.716
Survival	11,305 (46.4)	119,460 (74.3)		
Death	11,074 (45.4)	23,486 (14.6)		
Transfer to other institution	1,989 (8.2)	17,895 (11.1)		

ED = Emergency department; SD = Standard deviation

OHCA = Out of hospital Cardiac Arrest

ICU = Intensive care unit; ISS = Injury severity score; EMC = Emergency medical center

TAE = Trans-catheter arterial embolization

SMD = Standardized mean difference

### Inter-hospital transfer cohort

Table 4 summarizes inter-hospital transfer characteristics and receiving-hospital outcomes among transferred patients with available receiving-hospital outcome information. This subset should be interpreted descriptively, not as an analysis of all transferred patients or as a causal evaluation of transfer. Mechanism distribution and reasons for transfer differed by injury intent: intentional injuries more often involved drug intoxication or hanging/strangulation and were more frequently transferred to higher-level hospitals or because of bed unavailability, whereas unintentional injuries were mainly transportation- or fall-related and more often involved patient-related transfer reasons. At the receiving hospital, ED mortality, post-admission survival, and overall outcomes were broadly similar between groups.

**Table 4:** Inter-hospital transfer characteristics and receiving-hospital outcomes, by injury intent (*N* = 20,292)

	Intentional (*N* = 4,774)	Unintentional (*N* = 15,518)	*P*-value	SMD
**Mechanism of injury, N (%)**			< 0.001	3.079
**Traumatic injury**				
Transportation incidents	5 (0.1)	8,524 (54.9)		
Falls / Slip down	481 (10.1)	4,197 (27.0)		
Blunt trauma	173 (3.6)	436 (2.8)		
Stab/Penetrating trauma	344 (7.2)	111 (0.7)		
Machine-related injury	0 (0)	364(2.3)		
**Non-traumatic injury**				
Thermal injury	29 (0.6)	451 (2.9)		
Hanging/Strangling	611 (12.8)	19 (0.1)		
Drowning/Suffocation	26 (0.5)	505 (3.3)		
Drug intoxication	3,097 (64.9)	621 (4.0)		
Others	1 (0.0)	82 (0.5)		
Missing	7 (0.1)	208 (1.3)		
**Reason for Transfer**,** N (%)**			< 0.001	0.402
Lack of general ward, ICU beds	558 (11.7)	992 (6.4)		
Inability to provide care or surgery	406 (8.5)	1,474 (9.5)		
Transfer to a higher-level hospital	2,702 (56.6)	6,887 (44.4)		
Transfer to a lower-level hospital	73 (1.5)	576 (3.7)		
Transfer due to patient circumstances	1,007 (21.1)	5,532 (35.7)		
Others	28 (0.6)	57 (0.4)		
**ED disposition at the receiving hospital**,** N (%)**			< 0.001	0.235
Survival-to-discharge	592 (12.4)	892 (5.7)		
Survival-to-transfer	191(4.0)	679 (4.4)		
Survival-to-admission	3,785 (79.3)	13,314 (85.8)		
Death in the ED	206 (4.3)	633 (4.1)		
**ICU admission among** **admitted patients**,** N (%)**	2,492 (65.8)	7,722 (58.0)	< 0.001	0.162
**Result after admission**,** N (%)**	(*N* = 3,785)	(*N* = 13,314)	0.807	0.005
Survival to discharge	3,276 (86.6)	11,503 (86.4)		
Death	509 (13.4)	1,811 (13.6)		
**Result after ED**,** N (%)**			0.202	0.030
Survival	3,868 (81.0)	12,395 (79.9)		
Death	715 (15.0)	2,444 (15.7)		
Transfer to other institution	191 (4.0)	679 (4.4)		

ED = Emergency department; SMD = Standardized mean difference

### Primary multivariable logistic regression models in the non-intoxication cohort

Table 5 presents the primary multivariable logistic regression analysis of ED survival after excluding drug intoxication cases. The primary model adjusted for age group, sex, insurance status, broad injury mechanism category, and hospital level, without adjustment for ISS or OHCA. In this model, intentional injury was associated with substantially lower ED survival compared with unintentional injury (adjusted odds ratio [aOR], 0.215; 95% confidence interval [CI], 0.207–0.222; *P* < 0.001).

Compared with children aged 0–9 years, ED survival was lower in all older age groups, with progressively lower odds of ED survival among older patients. Female sex was associated with higher ED survival than male sex (aOR, 1.467; 95% CI, 1.423–1.513; *P* < 0.001). Medicaid coverage was associated with higher ED survival compared with medical insurance (aOR, 1.704; 95% CI, 1.593–1.823; *P* < 0.001), whereas other insurance types were associated with lower ED survival (aOR, 0.333; 95% CI, 0.315–0.352; *P* < 0.001). Non-traumatic injury was associated with higher ED survival compared with traumatic injury (aOR, 2.591; 95% CI, 2.496–2.690; *P* < 0.001). Compared with trauma centers, ED survival was lower among patients treated at regional emergency medical centers, local emergency medical centers, and emergency medical centers.

**Table 5:** Primary model for ED survival in the non-intoxication cohort (*N* = 183,045)

Variables	Adjusted OR	95% CI	*P*-value
**Injury intent**			
Unintentional injury	1		
Intentional injury	0.215	0.207–0.222	< 0.001
**Age, years**			
0–9	1		
10–19	0.363	0.318–0.415	< 0.001
20–29	0.350	0.309–0.396	< 0.001
30–39	0.300	0.265–0.340	< 0.001
40–49	0.260	0.231–0.294	< 0.001
50–59	0.220	0.196–0.248	< 0.001
60–69	0.190	0.169–0.214	< 0.001
70–79	0.146	0.130–0.165	< 0.001
80-	0.138	0.122–0.155	< 0.001
**Sex,**			
Male	1		
Female	1.467	1.423–1.513	< 0.001
**Insurance**			
Medical insurance	1		
Medicaid	1.704	1.593–1.823	< 0.001
Others	0.333	0.315–0.352	< 0.001
**Mechanism of injury**			
Traumatic injury	1		
Non-traumatic injury	2.591	2.496–2.690	< 0.001
**Level of hospital**			
Trauma center	1		
Regional EMC	0.730	0.695–0.768	< 0.001
Local EMC	0.718	0.687–0.750	< 0.001
EMC	0.526	0.502–0.551	< 0.001

ED = Emergency department; CI = Confidence Interval

EMC = Emergency medical center

Table 6 presents the primary multivariable logistic regression analysis of post-admission survival after excluding drug intoxication cases, using the same adjustment strategy. Intentional injury was associated with lower post-admission survival compared with unintentional injury (aOR, 0.561; 95% CI, 0.521–0.604; *P* < 0.001).

Compared with children aged 0–9 years, post-admission survival was not significantly different among patients aged 10–39 years, but decreased among older age groups, particularly among those aged 50 years or older. Female sex was associated with higher post-admission survival than male sex (aOR, 1.376; 95% CI, 1.303–1.453; *P* < 0.001). Medicaid coverage was associated with slightly higher post-admission survival compared with medical insurance (aOR, 1.133; 95% CI, 1.019–1.260; *P* = 0.021), whereas other insurance types were associated with lower post-admission survival (aOR, 0.580; 95% CI, 0.514–0.655; *P* < 0.001). Non-traumatic injury was associated with higher post-admission survival compared with traumatic injury (aOR, 3.294; 95% CI, 3.069–3.535; *P* < 0.001). Compared with trauma centers, treatment at regional emergency medical centers was not significantly associated with post-admission survival, whereas treatment at local emergency medical centers and emergency medical centers was associated with higher post-admission survival. These institution-level findings should be interpreted cautiously because they may reflect case mix, selection, and survivor effects rather than independent causal effects.

**Table 6:** Primary model for post-admission survival in the non-intoxication cohort (*N* = 65,719)

Variables	Adjusted OR	95% CI	*P*-value
**Injury intent**			
Unintentional injury	1		
Intentional injury	0.561	0.521–0.604	< 0.001
**Age, years**			
0–9	1		
10–19	1.031	0.815–1.304	0.798
20–29	1.024	0.826–1.270	0.829
30–39	1.031	0.832–1.278	0.779
40–49	0.817	0.665–1.004	0.054
50–59	0.697	0.570–0.852	< 0.001
60–69	0.526	0.431–0.643	< 0.001
70–79	0.357	0.293–0.436	< 0.001
80-	0.307	0.250–0.376	< 0.001
**Sex,**			
Male	1		
Female	1.376	1.303–1.453	< 0.001
**Insurance**			
Medical insurance	1		
Medicaid	1.133	1.019–1.260	0.021
Others	0.580	0.514–0.655	< 0.001
**Mechanism of injury**			
Traumatic injury	1		
Non-traumatic injury	3.294	3.069–3.535	< 0.001
**Level of hospital**			
Trauma center	1		
Regional EMC	0.942	0.882–1.006	0.076
Local EMC	1.168	1.101–1.239	< 0.001
EMC	2.754	2.497–3.038	< 0.001

CI = Confidence Interval; EMC = Emergency medical center

### Secondary and sensitivity models

In the ISS-adjusted secondary models restricted to patients with valid anatomical ISS values, intentional injury remained associated with lower ED survival (aOR, 0.398; 95% CI, 0.345–0.460; *P* < 0.001; Supplementary Table S2) and lower post-admission survival (aOR, 0.847; 95% CI, 0.752–0.954; *P* = 0.006; Supplementary Table S4). Higher ISS was also associated with lower ED survival (aOR, 0.914 per 1-point increase; 95% CI, 0.911–0.917; *P* < 0.001; Supplementary Table S2) and lower post-admission survival (aOR, 0.896 per 1-point increase; 95% CI, 0.894–0.899; *P* < 0.001; Supplementary Table S4).

In the sensitivity models additionally adjusting for prehospital OHCA without ISS, intentional injury remained associated with lower ED survival (aOR, 0.554; 95% CI, 0.518–0.593; *P* < 0.001; Supplementary Table S3) and lower post-admission survival (aOR, 0.809; 95% CI, 0.738–0.886; *P* < 0.001; Supplementary Table S5). These OHCA-adjusted sensitivity models used the same analytic framework as the corresponding primary models, with prehospital OHCA added as an additional covariate. Prehospital OHCA was strongly associated with lower ED survival (aOR, 0.002; 95% CI, 0.001–0.002; *P* < 0.001; Supplementary Table S3) and lower post-admission survival (aOR, 0.033; 95% CI, 0.029–0.036; *P* < 0.001; Supplementary Table S5).

Overall, intentional injury was consistently associated with lower survival in the ED and after hospital admission across the primary, ISS-adjusted secondary, and OHCA-adjusted sensitivity models.

### Age-specific incidence, case counts, case fatality, and mortality

**Fig. 2:**
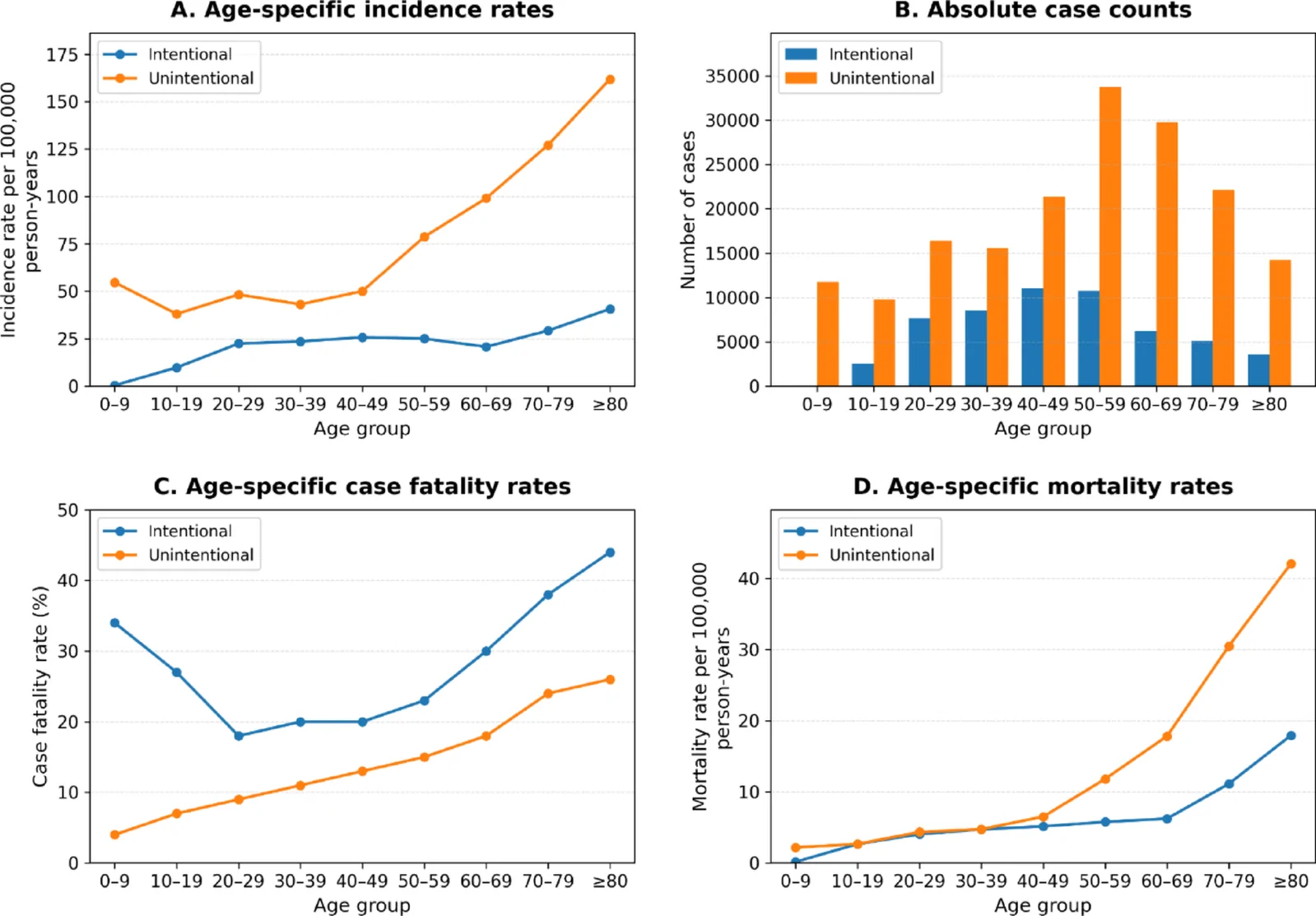
Age-specific distribution of intentional and unintentional severe external-cause injuries. (**A**) Age-specific incidence rates per 100,000 person-years during 2016–2020, calculated using summed annual resident population denominators. (**B**) Absolute case counts by age group. (**C**) Age-specific case fatality, defined as deaths divided by transported cases within each age group. (**D**) Age-specific mortality rates per 100,000 person-years during 2016–2020, calculated using the same summed annual resident population denominators

Figure 2 shows pooled 2016–2020 age-specific incidence rates and mortality rates per 100,000 person-years, absolute case counts, and age-specific case fatality according to recorded injury intent. Incidence and mortality rates were calculated using summed annual age-specific resident population denominators for 2016–2020, which are presented in Supplementary Table S6. In terms of age-specific incidence rates (Fig. 2A), unintentional injuries consistently showed higher rates than intentional injuries across all age groups, with a marked increase in older adults. Intentional injury incidence increased during young adulthood and showed an additional increase in older age groups.

Absolute case counts are shown in Fig. 2B. Unintentional injury cases were more numerous than intentional injury cases across age groups and were concentrated in middle-aged and older adults. Intentional injury cases were concentrated mainly in adult age groups.

Age-specific case fatality was higher for intentional injuries than for unintentional injuries across all age groups (Fig. 2C). However, the apparently high case fatality estimate in the 0–9-year intentional injury subgroup should be interpreted cautiously because the number of transported intentional injury cases in this age group was small. In contrast, case fatality for unintentional injuries increased progressively with age.

Age-specific mortality rates showed different patterns by injury intent (Fig. 2D). Unintentional injury mortality increased steadily with age and was generally higher than intentional injury mortality, particularly from middle age onward. Intentional injury mortality also increased with age, but remained lower than unintentional injury mortality in most age groups. Both injury types showed high mortality rates among older adults.

Population denominators were derived from the summed annual age-specific resident population counts from Statistics Korea for 2016–2020. Because the survey target population was limited to patients transported to emergency departments by 119 EMS, patients who died at the scene without EMS transport were outside the survey population.

### Regional variation in crude rate ratios

**Fig. 3:**
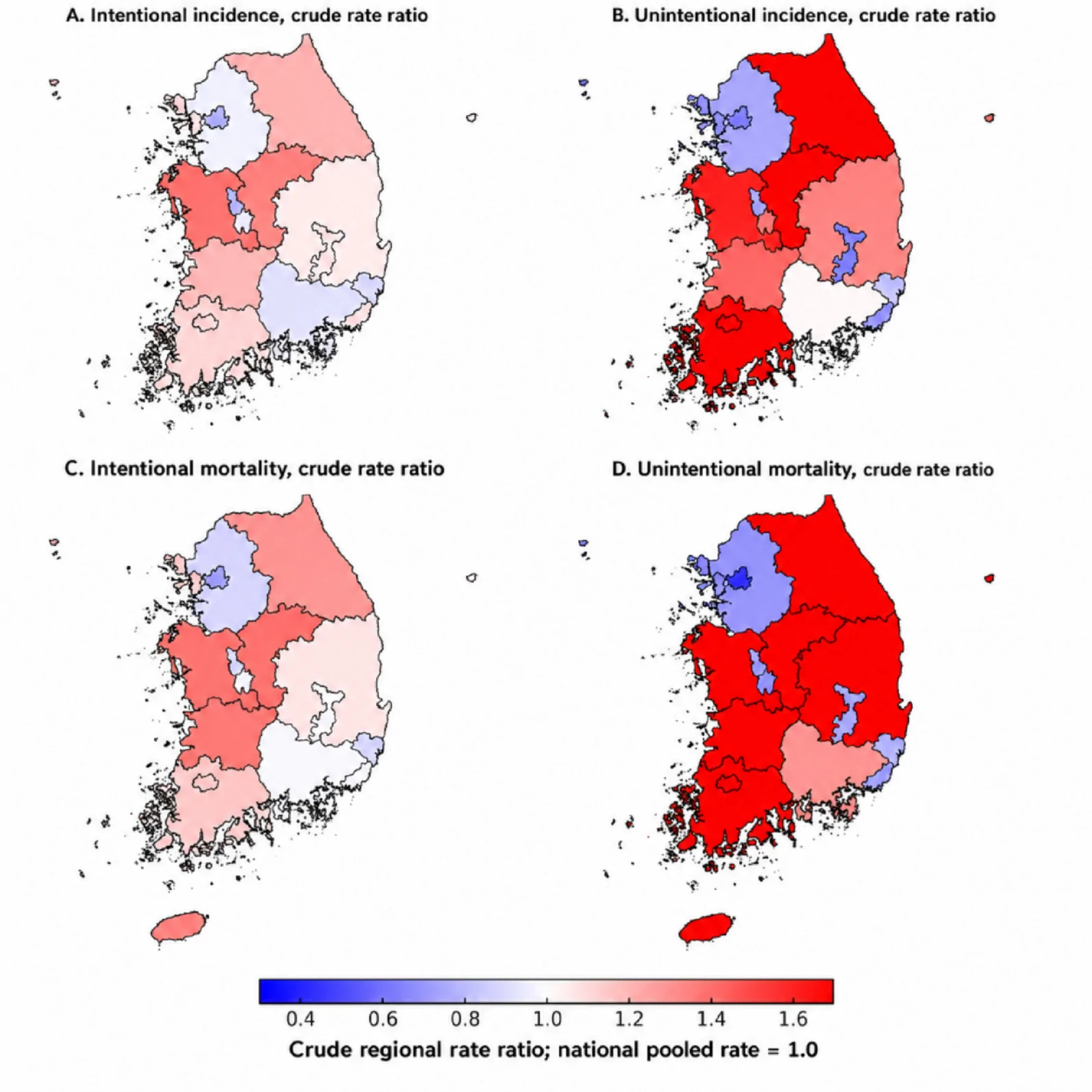
Regional variation in intentional and unintentional EMS-transported severe external-cause injuries in Korea. (**A**) Crude regional rate ratio for intentional injury incidence. (**B**) Crude regional rate ratio for unintentional injury incidence. (**C**) Crude regional rate ratio for intentional injury mortality. (**D**) Crude regional rate ratio for unintentional injury mortality

Figure 3 presents regional variation in intentional and unintentional EMS-transported severe external-cause injuries in South Korea. Regional differences are expressed as crude regional rate ratios, using the national pooled crude rate during 2016–2020 as the reference value of 1.0.

For intentional injury incidence, crude regional rate ratios were generally lower in metropolitan areas and relatively higher in several small- to medium-sized cities and rural regions. A similar descriptive pattern was observed for unintentional injury incidence, with lower crude rate ratios in metropolitan areas and higher crude rate ratios in many non-metropolitan regions.

For intentional injury mortality, crude regional rate ratios were also generally lower in metropolitan areas and higher in several non-metropolitan regions. Unintentional injury mortality showed a similar descriptive regional pattern, with higher crude rate ratios observed across many non-metropolitan regions. Because these estimates were crude and were not age- or sex-standardized, the regional findings should be interpreted as descriptive patterns rather than adjusted regional comparisons.

Regional differences are shown as crude regional rate ratios according to injury occurrence location. Regional crude rate ratios were calculated by dividing each region’s crude incidence or mortality rate by the national pooled crude rate during 2016–2020, with the national pooled rate used as the reference value of 1.0. Values are crude estimates and were not age- or sex-standardized.

## Discussion

In this nationwide EMS-based study of severe external-cause injuries in South Korea, we found substantial differences in demographic characteristics, mechanism distribution, prehospital deterioration, age-specific burden, regional patterns, transfer pathways, and stage-specific clinical outcomes according to recorded injury intent. This study adds to the existing literature by using a nationwide EMS-based dataset to evaluate differences according to recorded injury intent across multiple stages of acute care, including mechanism distribution, prehospital deterioration, inter-hospital transfer pathways, ED survival, and post-admission survival. Importantly, this study distinguished injury intent from injury mechanism, consistent with injury surveillance frameworks in which external-cause injuries are classified according to both mechanism and intent [2, 4]. Injury intent was based on the classification recorded in the Community-Based Severe Trauma Survey, whereas injury mechanism represented the external cause or clinical mechanism of injury [16, 17]. Therefore, the present analysis should be interpreted as a comparison of KDCA-defined EMS-transported severe external-cause injuries classified by recorded intent, rather than as a comparison limited to anatomically defined trauma.

In the non-intoxication cohort, injuries classified as intentional were associated with lower ED survival and lower post-admission survival than injuries classified as unintentional. In the primary multivariable models, intentional injury remained associated with lower ED survival and post-admission survival after adjustment for age group, sex, insurance status, broad injury mechanism category, and hospital level. These associations persisted in secondary models restricted to patients with valid anatomical ISS values and in sensitivity models additionally adjusting for prehospital OHCA. However, because the ISS-adjusted and OHCA-adjusted models differ in analytic sample and covariate structure, these results should be read as supporting robustness across frameworks rather than as a simple attenuation gradient.

The mechanism distribution differed markedly between injuries classified as intentional and those classified as unintentional. Among injuries classified as intentional, drug intoxication and asphyxial mechanisms, particularly hanging or strangulation, accounted for large proportions, whereas transportation-related incidents and falls predominated among injuries classified as unintentional. These findings support the epidemiologic value of classifying injuries according to both intent and mechanism. Mechanisms such as drug intoxication, hanging, strangulation, drowning, suffocation, falls, or penetrating injury are not synonymous with injury intent; rather, they may occur in either intentional or unintentional contexts depending on the circumstances of the event [2, 4]. Drug intoxication is a toxicologic mechanism that is not well represented by anatomical ISS; intoxication cases were therefore described separately and excluded from the primary non-intoxication survival models, and descriptive ISS summaries in the non-intoxication cohort should be read as anatomical injury severity only [18, 20]. 

The age-specific findings showed that the burden of EMS-transported severe external-cause injuries varied across the life course according to recorded intent. Injuries classified as unintentional showed higher pooled age-specific incidence rates across all age groups, with a pronounced increase among older adults. In the revised age-specific analysis, unintentional injury mortality increased steadily with age and was generally higher than intentional injury mortality, particularly from middle age onward. Intentional injury mortality also increased with age, but remained lower than unintentional injury mortality in most age groups. In contrast, case fatality among transported cases was higher for intentional injuries than for unintentional injuries across age groups. However, the apparently high case fatality estimate among children aged 0–9 years with intentional injuries should be interpreted cautiously because the number of transported intentional injury cases in this age stratum was small. These rates should be interpreted as the age-specific burden of EMS-transported severe external-cause injuries rather than the incidence of all injuries in the community, because they were calculated within the KDCA survey target population using summed annual resident population denominators [17, 21]. In South Korea, the age-specific pattern of injuries classified as intentional should be interpreted in the context of suicide and self-harm as major public health concerns, particularly among older adults [6]. Socioeconomic vulnerability may also be relevant. Patients classified as having intentional injuries more frequently had Medicaid coverage or other non-standard insurance types. Prior evidence has linked low socioeconomic status with suicidal ideation among older individuals [24]. However, insurance status should be interpreted only as a descriptive socioeconomic indicator because the dataset did not include individual-level information on psychiatric history, alcohol use, social isolation, household income, occupation, or prior self-harm. In addition, covariate-specific estimates for insurance status in the multivariable models should not be interpreted as independent causal effects, because these variables may reflect case mix, selection, and healthcare pathways. Therefore, these findings support the need for tailored prevention strategies but do not establish a causal socioeconomic pathway [25]. 

Injuries classified as intentional were associated with substantially worse early clinical outcomes than injuries classified as unintentional. After excluding drug intoxication cases, patients classified as having intentional injuries showed markedly higher rates of OHCA, ED death, and overall mortality. Previous studies have similarly reported that intentional traumatic injuries are associated with higher mortality, and intentional traumatic brain injury has been associated with greater injury severity and mortality [26, 27]. In the present study, however, valid anatomical ISS values were similar in absolute terms between the intentional and unintentional groups after excluding drug intoxication, whereas OHCA and ED mortality were substantially higher among patients classified as having intentional injuries. This finding suggests that anatomical injury severity alone did not fully explain the excess early mortality observed among injuries classified as intentional. ISS is designed to summarize structural injury burden and may not adequately capture early physiological deterioration related to delayed discovery, asphyxial mechanisms, toxicologic mechanisms, or other circumstances surrounding the injury event [18, 20]. 

Non-intoxication cohort findings also clarify the pattern of post-ED care. Among admitted patients, ICU admission was more frequent in the intentional injury group, whereas surgery or transcatheter arterial embolization was performed at nearly identical frequencies between intentional and unintentional injuries. This pattern suggests that, after excluding drug intoxication, intentional injuries were not simply characterized by lower use of procedural or surgical intervention. Rather, the excess risk associated with intentional injury appeared to be concentrated in early physiological deterioration, ED mortality, and subsequent post-admission survival, while operative or angiographic intervention rates among admitted patients were similar between groups.

The stage-specific outcome analyses further suggested that the survival disadvantage associated with intentional injury classification was most prominent before or during the ED phase. The primary models were not adjusted for ISS or prehospital physiological variables. Prehospital vital signs, mental status, and OHCA may lie on the pathway between the injury event, early physiological deterioration, EMS contact, and survival. Conversely, secondary ISS-adjusted models were used to evaluate whether the association persisted among patients with valid anatomical injury severity information, and sensitivity models additionally adjusting for OHCA were used to examine robustness under an alternative adjustment strategy. The ISS-adjusted models should therefore be interpreted as analyses restricted to patients with valid anatomical injury severity information, not as direct evidence that adjustment for ISS alone attenuated the association in the full non-intoxication cohort. Similarly, the OHCA-adjusted models should be interpreted as sensitivity analyses because OHCA is closely linked to ED death and may represent downstream physiological deterioration rather than a baseline confounder. The covariate-specific estimate for the broad non-traumatic mechanism category should also be interpreted cautiously because this category included heterogeneous mechanisms, including asphyxial and drowning-related events, for which anatomical injury severity and physiological deterioration may not align. Therefore, differences in the direction or magnitude of the non-traumatic mechanism estimate across the primary, ISS-adjusted, and OHCA-adjusted models should not be interpreted as a contradiction, but rather as a reflection of mechanism heterogeneity and differential conditioning on anatomical severity or prehospital physiological deterioration.

From a systems perspective, these findings suggest that EMS triage for intentional injuries, particularly asphyxial mechanisms, may need to consider early physiological deterioration and the receiving hospital’s critical care capacity in addition to anatomical injury severity alone. This implication should be interpreted cautiously because the present study did not directly evaluate specific prehospital interventions, airway management strategies, or destination protocols. Nevertheless, the combination of high OHCA frequency, high ED mortality, and increased ICU admission among admitted intentional injury patients suggests that early recognition of physiologic instability may be especially important in this group.

Sex differences should be interpreted in relation to analytic cohort and mechanism composition. In the full cohort, the sex distribution of injuries classified as intentional was nearly balanced, whereas unintentional injuries were predominantly male. After excluding drug intoxication, male patients accounted for a larger proportion of both intent groups, but the between-group difference was small. Previous suicide research has described the gender paradox, in which females more frequently report suicidal ideation or attempts, whereas males experience higher suicide mortality, partly because of differences in method lethality [28, 29]. However, this study included only EMS-transported severe external-cause injuries, and the sex distribution may therefore differ from that observed in general self-harm or community-based injury populations. In trauma populations, sex-based survival differences have also been inconsistent across studies [30, 31]. Therefore, sex differences in the present study should be interpreted as descriptive findings that may reflect mechanism composition, lethality, injury severity, prehospital deterioration, and selection into the EMS-transported severe injury cohort.

Injuries classified as unintentional were more common among males and increased markedly with age. This pattern may reflect greater exposure to transportation-related incidents, falls, occupational injuries, and environmental hazards, which are major contributors to the burden of unintentional injuries [32, 33]. These considerations may partly explain the higher burden of unintentional injuries among older adults and in several non-metropolitan regions. However, the present dataset did not directly measure occupation, transportation exposure, environmental hazards, or exposure intensity, and these interpretations should therefore be considered descriptive.

The transfer analysis provided descriptive information on system-level care pathways rather than causal evidence of transfer effects. Because Table 4 was restricted to transferred patients with available receiving-hospital outcome information, the transfer findings should be interpreted as receiving-hospital outcome patterns among an ascertainable transfer subset, not as outcomes for all transferred patients. Among transferred patients with available receiving-hospital outcome information, patients classified as having intentional injuries more frequently had non-traumatic mechanisms, particularly drug intoxication and hanging or strangulation, whereas patients classified as having unintentional injuries were more commonly affected by transportation incidents and falls. Patients classified as having intentional injuries were also more frequently transferred to a higher-level hospital or because of lack of available ward or ICU beds, whereas patient-related transfer reasons were more common among patients classified as having unintentional injuries. However, ED mortality, post-admission survival, and overall outcomes after presentation to the receiving hospital were similar between groups. These findings suggest that transfer patterns may reflect differences in case-mix, institutional capacity, bed availability, geographic accessibility, and regional resource distribution, but they should not be interpreted as evidence that transfer itself caused differences in survival. Previous Korean studies have reported survival benefits associated with trauma center care among selected severe injury populations, including intentionally self-injured patients [9, 10]. In addition, national efforts to develop regional trauma systems have aimed to reduce preventable trauma deaths in Korea [34, 35]. Nevertheless, hospital level and transfer status in the present study should be interpreted as markers of care pathways and system organization rather than isolated causal determinants of survival.

Regional variation was observed in crude regional rate ratios for both incidence and mortality. These crude rate ratios were generally lower in metropolitan areas and higher in several non-metropolitan regions for both injury intent groups. For injuries classified as intentional, regional differences may be related to variation in suicide risk, mental health access, social isolation, socioeconomic conditions, and local healthcare resources. Previous Korean research has reported differences in suicide rates according to urbanicity and spatial clustering of suicide mortality [36]. For injuries classified as unintentional, regional variation may reflect differences in transportation exposure, road environment, occupational structure, environmental hazards, safety resources, EMS accessibility, and access to specialized trauma or emergency care [32, 37]. However, the regional estimates in this study were crude regional rate ratios based on pooled regional rates and were not age- or sex-standardized. Therefore, the spatial findings should be interpreted as descriptive and hypothesis-generating rather than as adjusted regional comparisons.

### Study limitations

This study has several limitations. First, the cohort comprised only KDCA-defined EMS-transported severe injury and mass casualty cases and does not represent all injured patients in the community; accordingly, the age-specific incidence and mortality rates reflect the burden within the survey target population rather than population rates of all injuries [17]. In particular, patients who died at the scene without EMS transport were not captured, whereas cases transported in arrest were retained (ISS code 88). If rapidly fatal intentional injuries were disproportionately excluded before EMS transport, the observed association between intentional injury and lower survival may have been underestimated.

Second, injury intent was based on survey records and may be misclassified, and the injury mechanisms examined should not be equated with intent. Drug intoxication was described separately but excluded from the primary survival models, limiting their generalizability to the full external-cause injury population. The intentional group also combined self-harm/suicide and violence/homicide, which may differ in mechanism and prognosis but were not analyzed separately.

Third, the regression models have inherent constraints. The primary models deliberately omitted ISS and prehospital physiological variables as potential mediators, and the secondary ISS-adjusted analyses were restricted to patients with valid anatomical ISS values, so differences across models partly reflect sample restriction rather than covariate adjustment alone. Residual confounding by unmeasured factors such as psychiatric history and socioeconomic status cannot be excluded, and because multiple comparisons were not formally adjusted, individual P-values should be interpreted together with standardized mean differences and clinical relevance.

Fourth, the inter-hospital transfer and regional analyses are descriptive and hypothesis-generating: the transfer cohort was limited to patients with available receiving-hospital outcomes, which may introduce selection bias, and the regional rate ratios were crude and not age- or sex-standardized.

Finally, only short-term outcomes (ED and post-admission survival) were assessed; long-term mortality, functional and neurological recovery, psychiatric follow-up, and recurrent self-harm were not evaluated.

## Conclusion

In conclusion, among KDCA-defined EMS-transported severe external-cause injuries in South Korea, injuries classified as intentional and unintentional differed substantially in mechanism distribution, prehospital deterioration, age-specific burden, regional patterns, transfer pathways, and stage-specific outcomes. Drug intoxication represented an important but clinically distinct mechanism and should be distinguished from injury intent itself. In the prespecified non-intoxication cohort, intentional injury classification was associated with lower ED and post-admission survival, with consistent findings in secondary ISS-adjusted and OHCA-adjusted models. These findings highlight the importance of clear intent-mechanism classification, early recognition of high-risk severe external-cause injuries, optimized EMS destination and transfer pathways, and age- and region-specific prevention strategies.

## Supplementary Information

Supplementary Material 1

## Data Availability

The datasets analyzed during the current study were obtained from the Korea Disease Control and Prevention Agency (KDCA) Community-Based Severe Trauma Survey under license. Restrictions apply to the availability of these data, which were used with permission for the current study and are not publicly available. Data may be available from the corresponding author upon reasonable request and with permission from the KDCA.

## Abbreviations

aOR: Adjusted odds ratio
CI: Confidence interval
ED: Emergency department
EMS: Emergency Medical Services
ICU: Intensive care unit
ISS: Injury Severity Score
KDCA: Korea Disease Control and Prevention Agency
OHCA: Out-of-hospital cardiac arrest
SMD: Standardized mean difference
TAE: Transcatheter arterial embolization
