# Supplementary material for: A nationwide comparison of epidemiologic characteristics and outcomes between intentional and unintentional severe external-cause injuries in Korea, 2016–2020
Source: Inj Epidemiol. 2026 Jul 16;13:67. doi: 10.1186/s40621-026-00701-5 (PMC13651221; doi:10.1186/s40621-026-00701-5)
Supplement: Supplementary file 1 [file 40621_2026_701_MOESM1_ESM.docx]

**Supplementary Tables**

| **Supplementary Table S1 – Clinical characteristics and outcomes of patients with drug intoxication according to recorded injury intent (N = 45,093)** | | | |
| --- | --- | --- | --- |
|  | Intentional  (N = 31,145) | Unintentional  (N = 13,948) | P-value |
| **Age, years (Mean ± SD)** | 49.6 ± 18.3 | 48.0 ± 21.4 | <0.001 |
| **Sex, N (%)** |  |  | <0.001 |
| Male | 12,977 (41.7) | 7,398 (53.0) |  |
| Female | 18,168 (58.3) | 6,550 (47.0) |  |
| **Insurance, N (%)** |  |  | <0.001 |
| Medical insurance | 25,693 (82.5) | 12,676 (90.9) |  |
| Medicaid | 3,313 (10.6) | 831 (6.0) |  |
| Others  (e.g., self-pay, automobile insurance) | 2,139 (6.9) | 441 (3.2) |  |
| **Time taken from 119 call to the ED arrival, minutes (Mean ± SD)** | 34.1 ± 18.2 | 47.1 ± 34.9 | <0.001 |
| **Level of hospital, N (%)** |  |  | <0.001 |
| Trauma center | 4,728 (15.2) | 1,340 (9.6) |  |
| Regional EMC | 7,041 (22.6) | 2,407 (17.3) |  |
| Local EMC | 15,060 (48.4) | 6,072 (43.5) |  |
| EMC | 4,316 (13.9) | 4,129 (29.6) |  |
| **Pre-hospital OHCA, N (%)** |  |  | <0.001 |
| OHCA | 1,164 (3.7) | 416 (3.0) |  |
| No OHCA | 29,981 (96.3) | 13,532 (97.0) |  |
| **Outcome in the ED, N (%)** |  |  | <0.001 |
| Survival-to-discharge | 12,082 (38.8) | 10,147 (72.7) |  |
| Survival-to-transfer | 3,644 (11.7) | 741 (5.3) |  |
| Survival-to-admission | 14,177 (45.5) | 2,779 (19.9) |  |
| Death in the ED | 1,242 (4.0) | 281 (2.0) |  |
| **ICU admission among**  **admitted patients, N (%)** | 9,987 (70.4) | 1,117 (40.2) | <0.001 |
| **Result after admission, N (%)** | (N=14,177) | (N=2,779) | 0.095 |
| Survival to discharge | 13,541 (95.5) | 2,674 (96.2) |  |
| Death | 636 (4.5) | 105 (3.8) |  |
| **Result after ED, N (%)** |  |  | <0.001 |
| Survival | 25,623 (82.3) | 12,821 (91.9) |  |
| Death | 1,878 (6.0) | 386 (2.8) |  |
| Transfer to other institution | 3,644 (11.7) | 741 (5.3) |  |
|  |  |  |  |
| ED = Emergency department; SD = Standard deviation; OHCA = Out of hospital Cardiac Arrest; EMC = Emergency medical center | | | |

| **Supplementary Table S2 – ISS-adjusted secondary multivariable model for ED survival in the non-intoxication severe external-cause injury cohort (N = 153,371)** | | | |
| --- | --- | --- | --- |
| **Variables** | Adjusted OR | 95% CI | P-value |
| **Injury intent** |  |  |  |
| Unintentional injury | 1 |  |  |
| Intentional injury | 0.398 | 0.345-0.460 | <0.001 |
| **Age, years** |  |  |  |
| 0-9 | 1 |  |  |
| 10-19 | 0.385 | 0.209-0.711 | 0.002 |
| 20-29 | 0.410 | 0.228-0.739 | 0.003 |
| 30-39 | 0.379 | 0.210-0.683 | 0.001 |
| 40-49 | 0.305 | 0.171-0.542 | <0.001 |
| 50-59 | 0.290 | 0.164-0.513 | <0.001 |
| 60-69 | 0.215 | 0.122-0.379 | <0.001 |
| 70-79 | 0.125 | 0.071-0.220 | <0.001 |
| 80- | 0.088 | 0.050-0.156 | <0.001 |
| **Sex,** |  |  |  |
| Male | 1 |  |  |
| Female | 1.179 | 1.060-1.310 | 0.002 |
| **Insurance** |  |  |  |
| Medical insurance | 1 |  |  |
| Medicaid | 1.148 | 0.920-1.434 | 0.222 |
| Others | 0.661 | 0.515-0.848 | 0.001 |
| **Mechanism of injury** |  |  |  |
| Traumatic injury | 1 |  |  |
| Non-traumatic injury | 0.538 | 0.421-0.687 | <0.001 |
| **ISS** (1-point increase) | 0.914 | 0.911-0.917 | <0.001 |
| **Level of hospital** |  |  |  |
| Trauma center | 1 |  |  |
| Regional EMC | 0.312 | 0.266-0.365 | <0.001 |
| Local EMC | 0.344 | 0.296-0.399 | <0.001 |
| EMC | 0.584 | 0.476-0.717 | <0.001 |
| ED = Emergency department; CI = Confidence Interval  ISS = Injury Severity Score; EMC = Emergency medical center | | | |

| **Supplementary Table S3 – Sensitivity model additionally adjusting for prehospital OHCA for emergency department survival in the non-intoxication severe external-cause injury cohort (N = 183,045)** | | | |
| --- | --- | --- | --- |
| **Variables** | Adjusted OR | 95% CI | P-value |
| **Injury intent** |  |  |  |
| Unintentional injury | 1 |  |  |
| Intentional injury | 0.554 | 0.518-0.593 | <0.001 |
| **Age, years** |  |  |  |
| 0-9 | 1 |  |  |
| 10-19 | 0.555 | 0.446-0.691 | <0.001 |
| 20-29 | 0.604 | 0.496-0.736 | <0.001 |
| 30-39 | 0.585 | 0.482-0.711 | <0.001 |
| 40-49 | 0.600 | 0.497-0.724 | <0.001 |
| 50-59 | 0.498 | 0.414-0.599 | <0.001 |
| 60-69 | 0.399 | 0.331-0.480 | <0.001 |
| 70-79 | 0.287 | 0.238-0.346 | <0.001 |
| 80- | 0.218 | 0.179-0.264 | <0.001 |
| **Sex,** |  |  |  |
| Male | 1 |  |  |
| Female | 1.152 | 1.085-1.222 | <0.001 |
| **Insurance** |  |  |  |
| Medical insurance | 1 |  |  |
| Medicaid | 1.208 | 1.069-1.364 | 0.002 |
| Others | 0.453 | 0.406-0.505 | <0.001 |
| **Mechanism of injury** |  |  |  |
| Traumatic injury | 1 |  |  |
| Non-traumatic injury | 0.218 | 0.203-0.234 | <0.001 |
| **Level of hospital** |  |  |  |
| Trauma center | 1 |  |  |
| Regional EMC | 0.598 | 0.543-0.659 | <0.001 |
| Local EMC | 0.657 | 0.604-0.714 | <0.001 |
| EMC | 0.648 | 0.592-0.709 | <0.001 |
| **OHCA** |  |  |  |
| Non-OHCA | 1 |  |  |
| OHCA | 0.002 | 0.001-0.002 | <0.001 |
| ED = Emergency department; OHCA = Out of hospital Cardiac Arrest  EMC = Emergency medical center , | | | |

| **Supplementary Table S4 – ISS-adjusted secondary multivariable model for post-admission survival in the non-intoxication severe external-cause injury cohort (N = 62,578)** | | | |
| --- | --- | --- | --- |
| **Variables** | Adjusted OR | Confidence interval | P-value |
| **Injury intent** |  |  |  |
| Unintentional injury | 1 |  |  |
| Intentional injury | 0.847 | 0.752-0.954 | 0.006 |
| **Age, years** |  |  |  |
| 0-9 | 1 |  |  |
| 10-19 | 1.559 | 1.127-2.156 | 0.007 |
| 20-29 | 1.788 | 1.319-2.424 | <0.001 |
| 30-39 | 1.930 | 1.420-2.625 | <0.001 |
| 40-49 | 1.303 | 0.973-1.746 | 0.076 |
| 50-59 | 1.090 | 0.820-1.449 | 0.551 |
| 60-69 | 0.739 | 0.557-0.980 | 0.036 |
| 70-79 | 0.422 | 0.318-0.559 | <0.001 |
| 80- | 0.277 | 0.208-0.368 | <0.001 |
| **Sex** |  |  |  |
| Male | 1 |  |  |
| Female | 1.265 | 1.180-1.357 | <0.001 |
| **Insurance** |  |  |  |
| Medical insurance | 1 |  |  |
| Medicaid | 0.990 | 0.860-1.139 | 0.889 |
| Others | 0.450 | 0.383-0.528 | <0.001 |
| **Mechanism of injury** |  |  |  |
| Traumatic injury | 1 |  |  |
| Non-traumatic injury | 0.983 | 0.846-1.141 | 0.820 |
| **ISS** (1-point increase) | 0.896 | 0.894-0.899 | <0.001 |
| **Level of hospital** |  |  |  |
| Trauma center | 1 |  |  |
| Regional EMC | 0.719 | 0.662-0.781 | <0.001 |
| Local EMC | 0.725 | 0.672-0.782 | 0.002 |
| EMC | 1.244 | 1.085-1.427 | <0.001 |
| ISS = Injury Severity Score; EMC = Emergency medical center | | | |

| **Supplementary Table S5 – Sensitivity model additionally adjusting for prehospital OHCA for post-admission survival in the non-intoxication severe external-cause injury cohort (N = 65,719)** | | | |
| --- | --- | --- | --- |
| **Variables** | Adjusted OR | Confidence interval | P-value |
| **Injury intent** |  |  |  |
| Unintentional injury | 1 |  |  |
| Intentional injury | 0.809 | 0.738-0.886 | <0.001 |
| **Age, years** |  |  |  |
| 0-9 | 1 |  |  |
| 10-19 | 1.042 | 0.801-1.355 | 0.759 |
| 20-29 | 1.099 | 0.861-1.402 | 0.448 |
| 30-39 | 1.160 | 0.909-1.479 | 0.232 |
| 40-49 | 0.925 | 0.732-1.169 | 0.515 |
| 50-59 | 0.753 | 0.600-0.946 | 0.015 |
| 60-69 | 0.525 | 0.418-0.658 | <0.001 |
| 70-79 | 0.332 | 0.264-0.416 | <0.001 |
| 80- | 0.270 | 0.215-0.340 | <0.001 |
| **Sex,** |  |  |  |
| Male | 1 |  |  |
| Female | 1.360 | 1.281-1.445 | <0.001 |
| **Insurance** |  |  |  |
| Medical insurance | 1 |  |  |
| Medicaid | 1.071 | 0.950-1.208 | 0.262 |
| Others | 0.548 | 0.477-0.629 | <0.001 |
| **Mechanism of injury** |  |  |  |
| Traumatic injury | 1 |  |  |
| Non-traumatic injury | 0.932 | 0.833-1.042 | 0.215 |
| **Level of hospital** |  |  |  |
| Trauma center | 1 |  |  |
| Regional EMC | 0.983 | 0.914-1.056 | 0.633 |
| Local EMC | 1.211 | 1.135-1.292 | <0.001 |
| EMC | 2.991 | 2.681-3.336 | <0.001 |
| **OHCA** |  |  |  |
| Non-OHCA | 1 |  |  |
| OHCA | 0.033 | 0.029-0.036 | <0.001 |
| ED = Emergency department; OHCA = Out of hospital Cardiac Arrest  EMC = Emergency medical center | | | |

| **Supplementary Table S6 – Summed annual age-specific resident population denominators used for incidence and mortality rate calculations, Korea, 2016–2020** | |
| --- | --- |
|  |  |
| **Age, years** | **Summed annual resident population counts, 2016–2020** |
| 0-9 | 21,586,545 |
| 10-19 | 25,490,163 |
| 20-29 | 34,793,458 |
| 30-39 | 37,188,022 |
| 40-49 | 42,355,108 |
| 50-59 | 42,326,351 |
| 60-69 | 28,960,062 |
| 70-79 | 16,829,494 |
| 80- | 8,236,630 |
